# Exploring Compound Promiscuity Patterns and Multi-Target Activity Spaces

**DOI:** 10.5936/csbj.201401003

**Published:** 2014-01-29

**Authors:** Ye Hu, Disha Gupta-Ostermann, Jürgen Bajorath

**Affiliations:** a Department of Life Science Informatics, B-IT, LIMES Program Unit Chemical Biology and Medicinal Chemistry, Rheinische Friedrich-Wilhelms-Universität, Dahlmannstr. 2, D-53113 Bonn, Germany; † These authors contributed equally to this work

## Abstract

Compound promiscuity is rationalized as the specific interaction of a small molecule with multiple biological targets (as opposed to non-specific binding events) and represents the molecular basis of polypharmacology, an emerging theme in drug discovery and chemical biology. This concise review focuses on recent studies that have provided a detailed picture of the degree of promiscuity among different categories of small molecules. In addition, an exemplary computational approach is discussed that is designed to navigate multi-target activity spaces populated with various compounds.

## Introduction

Over the past decade it has been increasingly recognized that many pharmaceutically relevant compounds are promiscuous in nature [1–3] and that many drugs elicit their therapeutic effects -and undesired side effects- through polypharmacology [4, 5]. For a number of drugs that were originally considered to be target-selective or -specific, high degrees of promiscuity and ensuing polypharmacology have been shown to be responsible for their efficacy, with protein kinase inhibitors applied in oncology being a prime example [6]. In addition, polypharmacology also provides the basis for drug repurposing [7–9], another current topic of high interest in pharmaceutical research.

**Figure 1:**
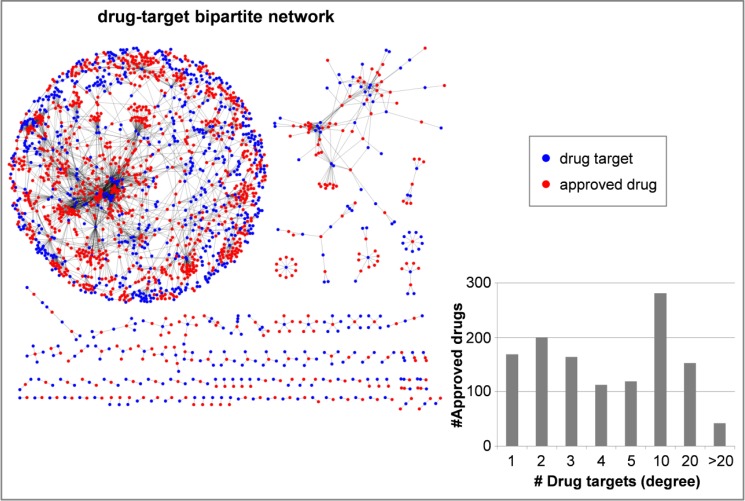
**Drug-target interactions**. Shown is an approved drug-target bipartite network. Red nodes represent approved drugs from DrugBank 3.0 and blue nodes drug targets. Edges between red and blue nodes indicate known drug-target interactions. In total, there are 3776 drug-target interactions between 1226 approved drugs and 881 targets. Similar yet distinct drug-based target networks have earlier been introduced by Yildirim et al. [29]. The insert reports the distribution of the degree of approved drug nodes, indicating the number of targets they were active against.

Given that compound promiscuity represents the molecular basis of polypharmacological effects, a detailed assessment of the degree of promiscuity among compounds at different stages of the drug development pathway is of considerable interest. The unprecedented recent growth of compound activity data in the public domain has made it possible to approach this question through data mining. This is illustrated in Figure 1, which shows a drug-target network generated on the basis of known target annotations of approved drugs, reflecting a generally high degree of drug promiscuity. In promiscuity analysis, most efforts have thus far concentrated on elucidating the promiscuous nature of drugs, often by database analyses combined with computational predictions. Recent estimates have been that a drug might on average interact with ∼3-6 targets and that 50% of all drugs might exhibit activity against more than five targets [5, 10].

Results of data mining efforts are generally affected by data incompleteness [10], i.e., not all compounds have been tested against all targets (and probably will never be). However, given increasingly large amounts of compound activity data that become available at present (much more than one could have imagined just a few years ago), reliable trends can already be detected and some meaningful conclusions drawn from them [11].

Herein, we review recent insights into promiscuity of screening hits, bioactive compounds, and drugs obtained through systematic mining of compound activity data. All currently investigated aspects of promiscuity are discussed. In addition, we introduce a computational and graphical framework for the analysis of multi-target activity spaces and compound promiscuity patterns.** The interested reader is also referred to other recent reviews of compound promiscuity [11, 12].

## Activity data of compounds from different sources

In order to comprehensively assess compound promiscuity, various types of compounds at different pharmaceutical development stages should be considered. A large number of relevant compounds and associated activity data can currently be collected from several public repositories.

### Screening hits

The PubChem BioAssay database [13] contains bioactivity information from confirmatory high-throughput screens including confirmed active and inactive compounds. To ensure high data confidence, a pre-requisite for meaningful data mining efforts [11], a total of 1085 confirmatory assays with reported activity against a single protein target and dose-response data were extracted from PubChem in January 2013 [14]. These assays involved 437,288 compounds and 439 targets.

A subset of 140,112 compounds was confirmed to be active in one or more assays, representing screening hits at the early stages of drug discovery. More than 77% of these hits were tested in more than 50 assays, hence providing a sound basis for promiscuity analysis [14], as discussed below.

### Bioactive compounds

The rapidly growing ChEMBL database [15] has become a major public repository of compound activity data obtained from medicinal chemistry sources. Currently, ChEMBL release 17 contains 1,324,941 distinct compounds with 12,077,491 activity annotations. It should be noted that the original investigations reviewed herein were carried out over time on different versions of ChEMBL (the versions were specified in each case).

To obtain high-confidence activity data from ChEMBL, only compounds with direct interaction against human targets at highest confidence level were extracted. Two types of potency measurements were separately considered, equilibrium constants (K_i_) and assay-dependent IC_50_ values. Compounds with approximate potency annotations (i.e., “ > ”, “ < ”, “∼”) were excluded. From ChEMBL release 14, 36,542 compounds active against 579 targets were collected that yielded 62,913 explicit K_i_ values, comprising the K_i_ subset. In the IC_50_ subset, there were 80,522 compounds active against 1129 targets with 114,092 IC_50_ measurements [16]. These bioactive molecules, especially those from the K_i_ subset, were predominantly taken from medicinal chemistry literature and patent sources and hence mostly represented compounds at the hit-to-lead and lead optimization stages.

### Experimental and approved drugs

The DrugBank database [17] is a public resource that contains drug entries, including approved small molecule drugs, approved biologicals, nutraceuticals, and experimental drugs (including compounds in clinical trials), with associated drug target information. For promiscuity analysis, 1274 approved small molecule drugs and 4931 experimental drugs with available structures were assembled from DrugBank 3.0. These approved drugs and drug candidates represented compounds at the late drug development stages.

## Compound promiscuity rates

From these different data repositories, promiscuous compounds were extracted and promiscuity rates calculated as the average number of targets compounds were active against. In all cases reported herein, promiscuity rates were determined for compounds active against multiple targets, i.e., excluding compounds with reported single-target activity. Taking compounds with single-target activity into account would have reduced average promiscuity rates.

**Figure 2:**
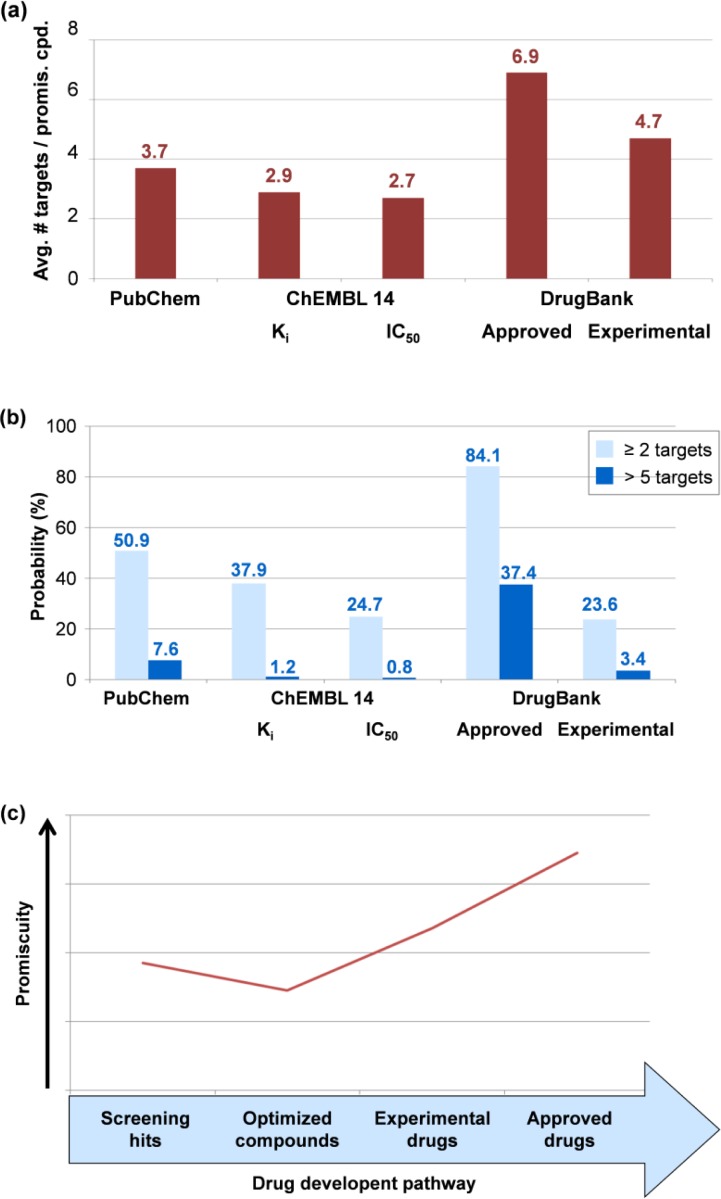
**Compound promiscuity rates**. (**a**) Reported is the average number of targets for promiscuous compounds from different sources. (**b**) Probability of compounds to be active against at least two (light blue) or more than five (dark blue) targets. (**c**) Relative promiscuity rates of compounds along the drug development pathway.

From 140,112 PubChem screening hits, 71,303 compounds (∼50.9%) were identified to be active against two or more targets [14]. In addition, for the K_i_ and IC_50_ subsets of ChEMBL version 14, 13,842 (∼37.9%) and 19,898 compounds (∼24.7%) were identified to be promiscuous, respectively [16]. These compounds were active against a total of 459 and 867 human targets in the K_i_ and IC_50_ subsets, respectively. Furthermore, compound overlap between these two subsets was established on the basis of database IDs. There were 1025 promiscuous compounds conserved in both subsets. The remaining 12,817 and 18,873 promiscuous compounds were exclusively found in the K_i_ and IC_50_ subsets, respectively. In general, the IC_50_ subset contained > 6000 more promiscuous compounds than the K_i_ subset. Furthermore, 1072 approved (∼84.1%) and 1113 experimental (∼23.6%) drugs from DrugBank had multiple target annotations. For compounds from different sources, promiscuity rates are reported in Figure 2a. On average, promiscuous compounds from PubChem confirmatory assays were active against 3.7 targets. Bioactive compounds from the K_i_ and IC_50_ subsets of ChEMBL interacted with 2.9 and 2.7 targets, respectively. Approved and experimental drugs displayed the highest degree of promiscuity, i.e., they had 6.9 and 4.7 targets, respectively [12].

Furthermore, from the distribution of promiscuity rates, the probability of compounds to be active against at least two or more than five targets was calculated [12]. The results are reported in Figure 2b. For screening hits, the probability to act against two or more targets was ∼50%. However, the probability of activity against more than five targets was reduced to 7.6%. For compounds from K_i_ and IC_50_ subsets of ChEMBL 14, the probability to interact with two or more targets was ∼38% and ∼25%, respectively. However, the probability of activity against more than five targets was reduced to only ∼1% for both subsets. For approved and experimental drugs, the probability of activity against two or more targets was ∼84% and ∼24% and the corresponding probability of activity against more than five targets ∼37% and ∼3%, respectively [12].

Taken together, the results indicated that the degree of promiscuity of bioactive compounds from screening or medicinal chemistry sources was considerably lower than for drugs. Thus, along the drug development pathway, a notable increase in promiscuity was observed from screening hits and optimized compounds over drug candidates to approved drugs, as illustrated in Figure 2c. These findings raise questions for further analysis. For example, do these observed differences mean that promiscuous drug candidates are preferentially selected during clinical trials? Or are target activities of drugs or drug candidates much more thoroughly assessed than those of other bioactive compounds? These alternative possibilities cannot be distinguished at present. It is evident, however, that bioactive compounds from various sources including high-throughput screens have a much lower degree of promiscuity than drugs on the basis of currently available data.

## Promiscuity across different target families

**Figure 3:**
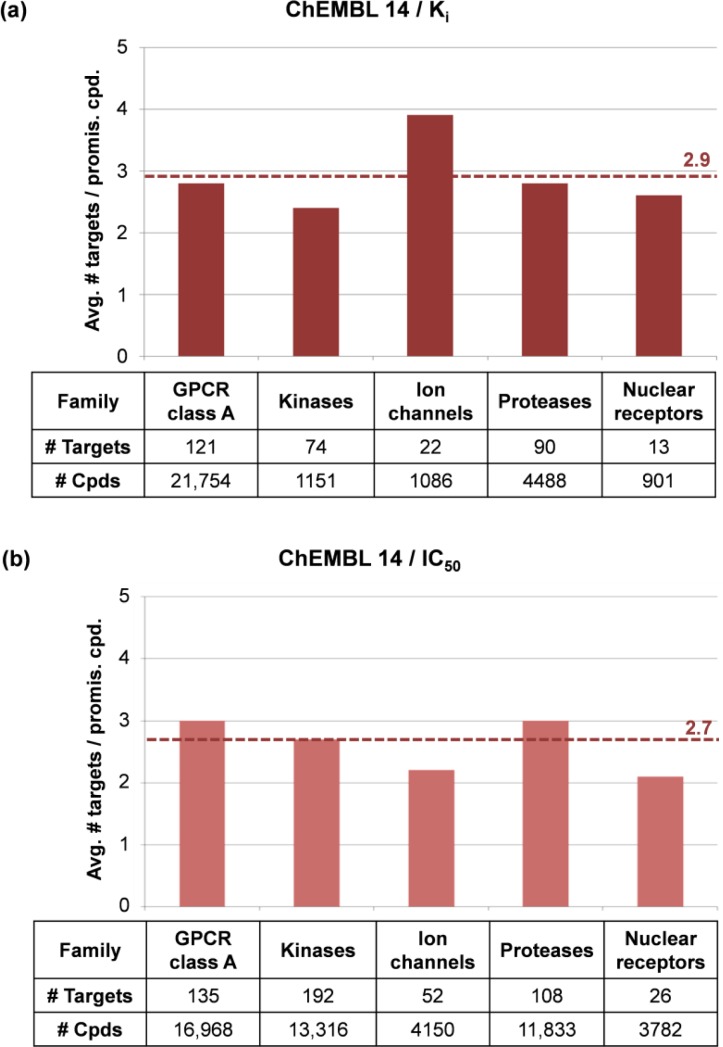
**Promiscuity across target families**. In (**a**) and (**b**), average promiscuity rates are reported for all compounds active against multiple targets within a given family for the K_i_ and IC_50_ subsets from ChEMBL 14, respectively. Dashed lines indicate global promiscuity rates determined for the K_i_ (i.e., on average 2.9 targets per compound) or IC_50_ subset (i.e., 2.7). For each target family, the number of targets and available active compounds is reported.

Compounds active against prominent therapeutic target families such as G-protein coupled receptors (GPCRs) or protein kinases have previously been reported to frequently exhibit high levels of promiscuity [1, 18]. Recently, compounds active against targets belonging to five different families were assembled from ChEMBL 14 including ligands of class A GPCRs, protein kinases, ion channels, proteases, and nuclear hormone receptors [12]. Compounds active against individual target families were further separated into K_i_ and IC_50_ value-based subsets. Average promiscuity rates of compounds active against multiple targets within a family were determined, as reported in Figure 3. For the K_i_-based subset, only compounds active against multiple ion channels displayed above-average promiscuity, with activity against 3.9 different channels (Figure 3a). By contrast, degrees of promiscuity for compounds active against the other four families were comparable to the global promiscuity rate determined for the entire K_i_ subset of ChEMBL 14, as discussed above. For the IC_50_-based subset, a different distribution of promiscuity rates was observed across these five target families. Compounds active against GPCR class A family and proteases showed a slightly higher than average degree of promiscuity (Figure 3b). However, the promiscuity rate of ion channel ligands was in this case lower than the global rate. Taken together, the results revealed no significant and consistent increase in promiscuity for compounds active against prominent target families relative to average promiscuity rates for bioactive compounds [12].

## Promiscuity vs. molecular weight

**Figure 4:**
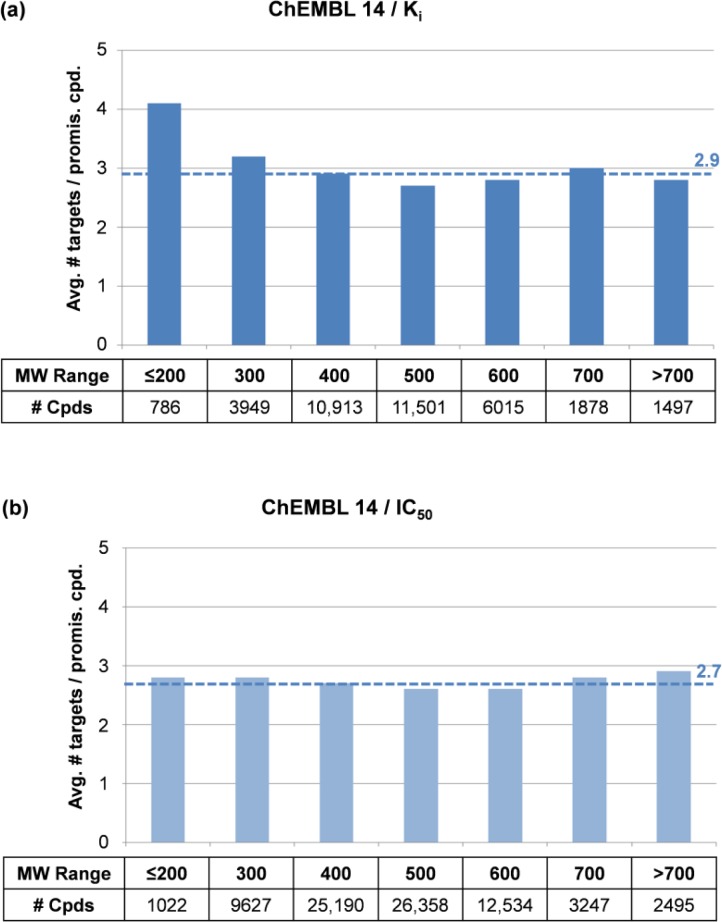
**Promiscuity vs. molecular weight**. In (**a**) and (**b**), promiscuity rates are reported for compounds classified by increasing molecular weight (MW) from the K_i_ and IC_50_ subsets of ChEMBL 14, respectively. Dashed lines indicate global promiscuity rates according to Figure 3.

Molecular complexity and size have frequently been implicated in promiscuity [19, 20]. Small compounds were found to display a general tendency to be more promiscuous than larger, chemically more complex molecules. A possible explanation for these findings is that small compounds and molecular fragments are easier to accommodate in differently shaped binding sites than larger ones. The relationship between compound promiscuity and molecular weight (MW) has also been systematically investigated through data mining [12]. Seven subsets of bioactive compounds with increasing (MW) were collected from ChEMBL 14. These compound subsets were also separated into K_i_ and IC_50_ value-based subsets. Figure 4 reports the compound composition of each MW range-based subset and the average promiscuity rates. For compounds with K_i_ values (Figure 4a), the subset of smallest compounds with MW of at most 200 Da displayed the highest degree of promiscuity with on average 4.1 targets per compound. Compounds with MW in the range of 200 to 300 Da had only slightly above-average promiscuity. For compounds with MW of more than 300, the degree of promiscuity was comparable to the global promiscuity rate for bioactive compounds. For compounds from the IC_50_ subset, there was even less variation over different MW ranges and all rates were close to the average promiscuity for IC_50_ data (Figure 4b). Therefore, with the exception of the smallest compounds with available K_i_ data, the degree of promiscuity did not notably depend on molecular size [12].

## Activity measurement dependence

**Figure 5:**
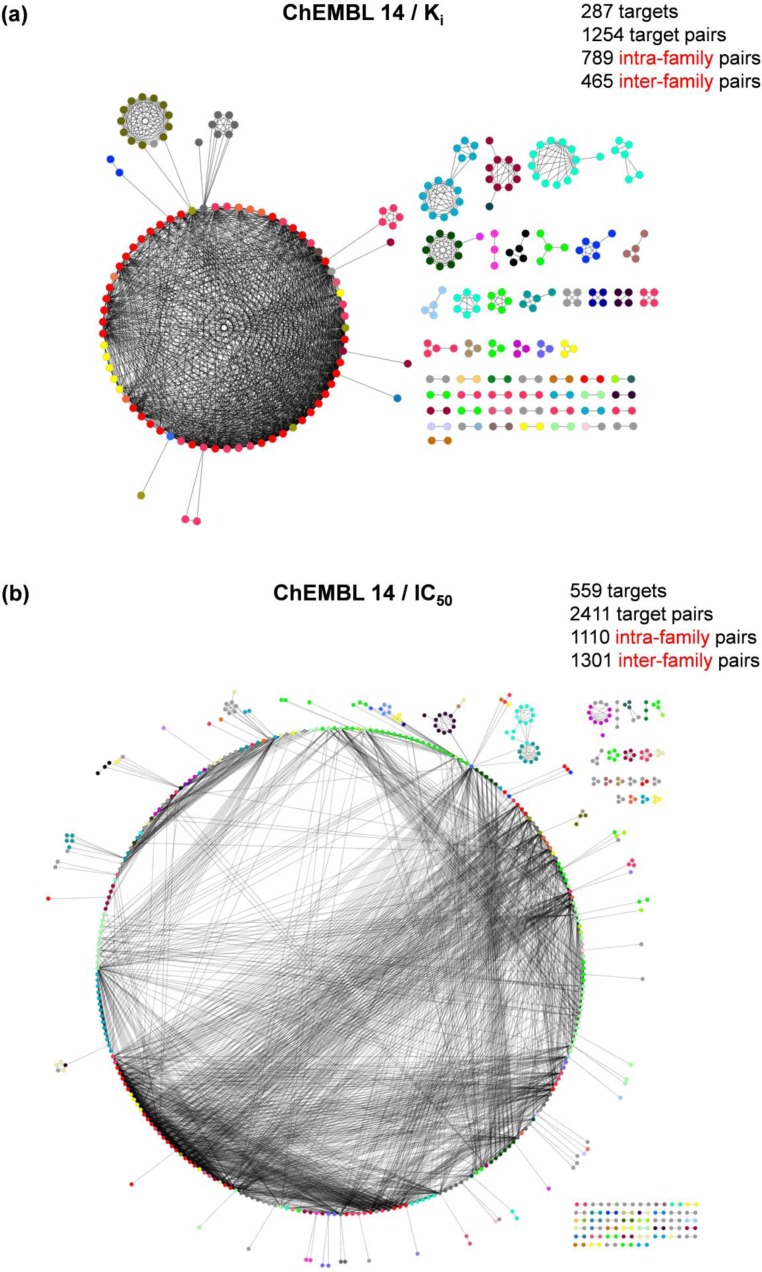
**Promiscuity-based target networks**. In (**a**) and (**b**), target networks are shown that are based on promiscuous compounds from the K_i_ and IC_50_ subsets of ChEMBL 14, respectively. Nodes represent targets (colored by target family) that are connected by an edge if they share at least five compounds. For each network, the number of nodes and edges are reported as well as the number of pairs formed by targets within the same family (intra-family pairs) or across different families (inter-family pairs). The layout of the target network reflects the connectivity or relationships between targets on the basis of at least five shared compounds. The length of the edges does not indicate the distance or the degree of relatedness between targets.

On the basis of global promiscuity rates determined for compounds from the K_i_ and IC_50_ subsets of ChEMBL, there was no significant difference between the degrees of promiscuity when these two different types of activity measurements were considered. The promiscuity rate was only slightly higher for compounds in the K_i_ than the IC_50_ subset (Figure 2a). However, when the original release of the ChEMBL database was compared with subsequent releases of ChEMBL up to version 13, it was also observed that the number of promiscuous compounds significantly increased over time. This increase was largely due to compounds with assay-dependent IC_50_ measurements, rather than equilibrium constants (K_i_) [21]. To further analyze this relative increase, compound-based target relationships were determined and visualized in network representations for two subsets of promiscuous compounds with available K_i_ (13,842 compounds) or IC_50_ measurements (19,898). The networks are shown in Figure 5. In each network, nodes represent targets that are connected by an edge if two targets share at least five compounds. In the K_i_ subset, a total of 1254 target pairs were formed that involved 287 targets. 789 pairs (∼63%) were formed by targets from the same family (intra-family pairs) and 465 pairs by targets from different families (inter-family pairs). The majority of the inter-family pairs formed a central network component (Figure 5a). The target network of the IC_50_ subset was clearly dominated by a single large component involving targets from many different families (Figure 5b). In this case, 2411 target pairs were formed involving 559 targets and ∼46% of the pairs were intra-family pairs. However, more than half of the pairs (∼54%) were formed across different target families. Thus, IC_50_ data yielded a significant increase in compound promiscuity across different target families.

## Structure-promiscuity relationships

Compound profiling data sets are obtained by screening compound libraries against arrays of targets. Currently, there are only few profiling data sets available in the public domain (most profiling data are produced in the pharmaceutical industry and kept proprietary). For example, Clemons and colleagues generated a small molecule microarray data set [22] using a total of 15,252 compounds assembled from diverse chemical sources including compounds from medicinal chemistry vendors, natural products, and compounds from diversity-oriented synthesis. These compounds were systematically screened against 100 sequence-unrelated proteins, i.e., a diverse spectrum of targets [22]. The experimentally determined activity data were then reported as a complete binary (active/inactive) matrix. Such data sets provide an opportunity to systematically explore structure-promiscuity relationships and structural determinants of promiscuity.

**Figure 6:**
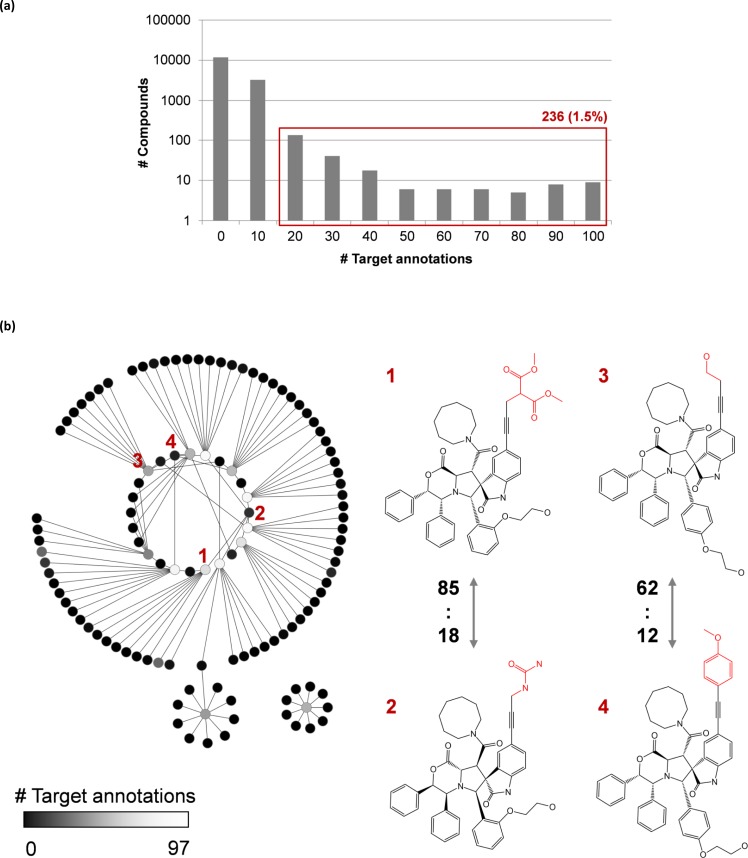
**Promiscuity cliffs**. (**a**) Distribution of target activities for compounds in a small molecule microarray data set. Compounds active against more than 10 targets are highlighted using a red box (236 compounds; ∼1.5% of the data set). (**b**) 126 promiscuity cliffs are organized in a network representation (left). Nodes represent compounds and edges indicate promiscuity cliffs. Nodes are colored according to the number of target activities using a continuous color spectrum from black (i.e., 0; inactive compounds) to white (i.e., 97; highest degree of promiscuity in the data set). Two representative promiscuity cliffs involving four compounds are shown (right). Structural differences are highlighted in red. For each compound, the number of targets is reported it was active against under microarray conditions.

For compounds comprising the microarray data set, the distribution of target annotations is reported in Figure 6a. The majority of compounds (i.e., 11,819; ∼77.5%) were inactive. The remaining compounds were active against 1-97 targets. However, only 236 compounds (∼1.5%) had activity against more than 10 targets. Therefore, highly promiscuous compounds were also rarely observed in the microarray experiment.

For analyzing structure-promiscuity relationships, the matched molecular pair (MMP) formalism was applied [23]. An MMP represents a pair of compounds that only differ at a single site by the exchange of two substructures, i.e., a chemical transformation. The application of transformation size restrictions typically limits substructure exchanges to chemically meaningful replacements [24]. From the entire microarray set, a total of 30,954 transformation size-restricted MMPs (i.e., ∼0.03% of all possible compound pairs) were obtained. Only a small subset of 126 MMPs was formed by compounds with large differences in the number of target annotations (50 or more targets) [25]. These MMPs represented small structural modifications leading to large-magnitude changes in promiscuity under the experimental conditions of the microarray experiment. The compound pairs were thus termed “promiscuity cliffs” [25] and are organized in a network representation in Figure 6b. In the network, nodes represent compounds and edges indicate the formation of promiscuity cliffs. The topology of the network reveals a number of “promiscuity hubs”, i.e., compounds involved in multiple promiscuity cliffs. Two representative promiscuity cliffs are also shown in Figure 6b. However, no chemical transformations or individual structural fragments were identified in the microarray data set that consistently introduced promiscuity cliffs or were exclusively present in highly promiscuous compounds. Large-magnitude changes in promiscuity might at least in part be triggered by experimental conditions of the microarray analysis. Nevertheless, the identified promiscuity cliffs provide interesting opportunities for follow-up investigations to explore potential structural determinants of compound promiscuity.

## Graphical mining of multi-target activity spaces

The analysis of multi-target spaces is a complex task but of high interest for compound design and development. For example, one would like to rationalize promiscuity patterns in compounds sets, explore structure-promiscuity relationships, and identify key compounds for further chemical exploration. Deconvoluting multi-target activity spaces also helps to investigate relationships between selective and promiscuous compounds. In the following, we introduce a computational methodology designed for mining multi-target activity spaces and visualizing promiscuity patterns, with a special focus on closely related compound series (currently, there are no other comparable approaches available).

### Compound Series Matrix

**Figure 7:**
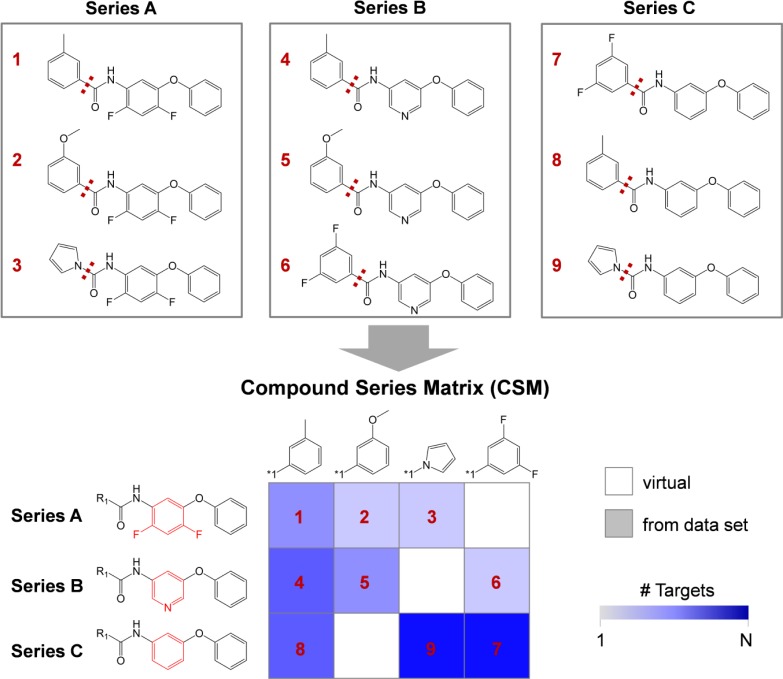
**Compound series matrix**. Three compound series (A, B and C) with related core structures resulting from MMP calculations are shown at the top. Each series contains three compounds that share a core structure (bottom left) and differ by small substituents. Structural differences between core structures are highlighted in red. The compound series matrix (CSM) is generated by combining structurally analogous series. Rows represent series and columns substituents. Each combination of a given core and substituent defines a real (filled cell) or virtual (empty cell) compound. Cells are colored according to the number of targets compounds are active against, hence reflecting the degree of compound promiscuity.

A data structure termed Compound Series Matrix (CSM) [26] was designed on the basis of the MMP formalism [23] to organize compound series with closely related core structures in multi-target space and elucidate promiscuity patterns. The CSM represents a methodological extension of the SAR matrix data structure previously introduced by us to monitor potency distributions of analogs active against a single target [27]. An analog series consists of a set of compounds that share the same core structure and differ by defined chemical substitutions (R-groups). CSMs utilize the same structural organization scheme as SAR matrices but take multi-target activities into account. Figure 7 illustrates the generation of a CSM. At the top, three analog series A, B, and C are shown that result from the application of a two-step MMP generation procedure following the fragmentation and indexing method of Hussain and Rea [23]. In the first step, MMPs are generated from original compounds. In the second step, MMPs are computed from the core fragments obtained in the first step. Thus, the second step produces MMPs with core structures that are only distinguished by a structural change at a single site. Therefore, the resulting analog series A, B, and C have structurally related cores and overlapping sets of substituents. The two-step fragmentation and MMP generation scheme is an essential feature of the methodology (further fragmentation steps cannot be applied to capture close and chemically meaningful structural relationships). The matrix is then filled with the core and substituent combinations, as illustrated at the bottom of Figure 7. Each related core structure represents a row and each substituent a column. Thus, compounds in a column share the same substituent and compounds in a row the same core structure. Each cell in the CSM represents a unique compound. Combinations of core structures and R-groups that are not present in the compound data set yield virtual matrix compounds from which candidates for synthesis can be selected. A color code is introduced to account for multi-target activities. If a compound is present in the data set it is colored using a spectrum from light blue to dark blue depending on the number of targets the compound is active against. Thus, CSMs establish structural relationships between compounds in multi-target activity space, capture promiscuity patterns in structurally related series, and provide hypotheses for compound design.

### CSM statistics

To evaluate the CSM methodology, compounds with reported K_i_ values of at least 10 µM (≤10 µM) for human targets were assembled from ChEMBL version 15. A total of 37,850 compounds were obtained that were active against 342 targets. The number of target annotations per compound ranged from 1 to 35. This pool of compounds was subjected to two-step MMP and CSM generation, yielding 2,337 different CSMs, 1665 of which contained promiscuous compounds. 1064 of these multi-target CSMs exclusively covered compounds active against targets from the same family, whereas the remaining 591 matrices contained compounds with activity against targets from 2 to 11 different families [26].

Promiscuity patterns

**Figure 8:**
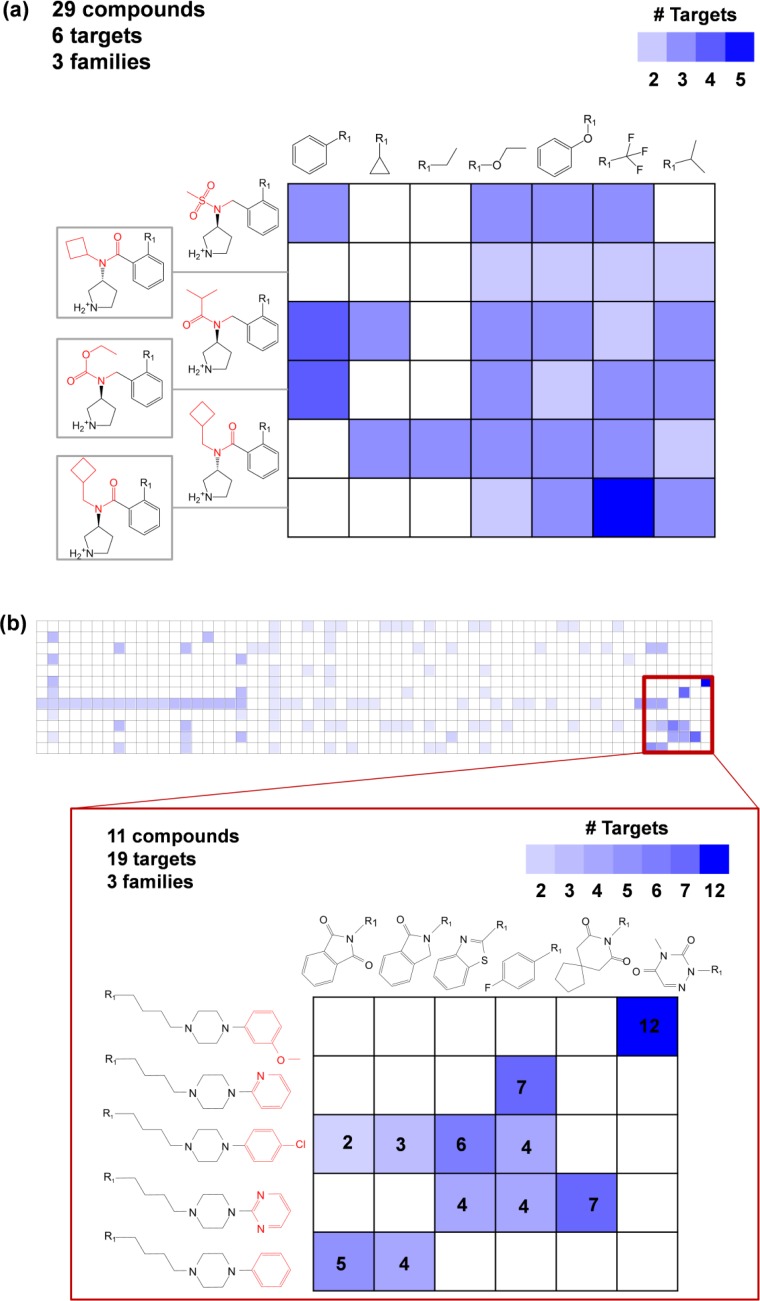
**Multi-target compound series matrices**. (**a**) Shown is a multi-target CSM containing 29 compounds active against six targets from three families. Structural differences between cores are highlighted in red. (**b**) A large CSM is shown that consists of 123 compounds active against 20 targets from four families. A region enriched by highly promiscuous compounds is highlighted and enlarged. Core structures and substituents are displayed. Taken together, the 11 compounds in this region are active against 19 targets from three families.

In Figure 8, two exemplary multi-target CSMs are shown that reveal compound promiscuity patterns. In Figure 8a, 29 compounds are represented by six related core structures and seven substituents. These compounds were active against six targets belonging to three different families. The number of targets per compound ranged from two to five. In the CSM, compounds sharing the same cores (rows) or substitutions (columns) displayed different degrees of promiscuity. Additionally, compounds with related cores and corresponding substitutions also displayed varying promiscuity. In Figure 8b, the most promiscuous matrix subset of a large and sparsely populated CSM comprising 123 compounds (top) is shown in detail (bottom). This subset contains 11 compounds represented by five related core structures and six substituents. The cores differ by aromatic ring substitutions highlighted in red. These compounds were active against a total of 19 different targets belonging to three different families. The compound in the top right cell was active against 12 targets of the monoamine GPCR family. As a compound design hypothesis, virtual compounds in this column provide suggestions for other compounds that might have a similar promiscuity profile. Hence, CSMs monitor promiscuity profiles of structurally related compound series at high resolution and contain many virtual entities that can be considered as candidates for the design of compounds with desired target profiles.

## Conclusion

Herein we have reviewed currently available insights into compound promiscuity obtained by systematic mining of activity data. In general, bioactive compounds from different sources including high-throughput screening and medicinal chemistry have a lower degree of promiscuity than indicated for drugs. In addition, there is relatively little variation of compound promiscuity for prominent drug target families when high-confidence activity measurements are considered. However, the degree of compound promiscuity across different target families is dependent on the types of activity measurements that are considered. This might result from more frequent determination of IC_50_ values of active compounds and diverse targets than equilibrium constants, which require larger experimental efforts. At the same time, it can also not be ruled out that assay promiscuity (rather than “true” target promiscuity) is at least partly responsible for rapidly increasing levels of cross-family promiscuity on the basis of IC_50_ data. Regardless, we emphasize that bioactive compounds display lower degrees of promiscuity on the basis of currently available data than often thought.

The degree of promiscuity of drugs is generally higher than promiscuity among screening hits and bioactive compounds, consistent with the emerging theme of drug polypharmacology. However, despite the increasing notion of polypharmacological drug actions, drug development in certain therapeutic areas will continue to focus on target-specific compounds [28]. Hence, reaching a balance between compound promiscuity and target specificity will likely be an important task for future drug discovery efforts [28].

As a computational and graphical framework to structurally organize compound data sets, navigate multi-target activity spaces, and visualize promiscuity patterns, the CSM approach has been discussed. CSMs not only enable the systematic study of compound promiscuity patterns but also support the exploration of novel compounds with desired target profiles, thereby integrating data mining and compound design.

## **

This review is based upon the presentation *‘Compound Data Mining in Systems Chemical Biology: Exploring Multi-target Activity Spaces and Compound Promiscuity Patterns’* given by one of us (J.B.) at the 2013 Meeting of the International Chemical Biology Society (ICBS2013). The program of ICBS2013 can be viewed via the following URL: http://www.chemical-biology.org/?page=ICBS2013Schedule).
